# Population Genomic Structure and Demographic History of Black Guillemots Breeding Across the North Atlantic

**DOI:** 10.1002/ece3.73126

**Published:** 2026-02-24

**Authors:** Bronwyn A. S. Harkness, Lila Colston‐Nepali, Gregory J. Robertson, Jennifer F. Provencher, Christopher K. Boccia, Vicki L. Friesen

**Affiliations:** ^1^ Department of Biology Queen's University Kingston Ontario Canada; ^2^ Wildlife Research Division Environment and Climate Change Canada Ottawa Ontario Canada; ^3^ Ecotoxicology and Wildlife Health Division Environment and Climate Change Canada Ottawa Canada

**Keywords:** *Cepphus*, conservation genomics, evolutionary history, population genetic structure, seabird

## Abstract

Identifying genetically differentiated populations is important for successful species conservation and management, and collecting baseline population genomic data can allow us to quantify impacts from environmental changes and anthropogenic stressors. Unlike most auks, which breed in a few large colonies, black guillemots ( *Cepphus grylle* ) are dispersed breeders, whose range spans diverse environmental conditions, from polar to temperate waters. They are harvested in some northern regions and can be an important indicator of coastal ecosystem health, but knowledge of their population genetic structure is limited. We used double‐digest restriction‐site associated DNA sequencing to determine the extent to which regional samples of black guillemots (*n =* 172) in the Arctic and North Atlantic oceans differ at presumptively neutral markers. Population genetic analyses identified three genetic clusters: (1) Northwest Atlantic: Gulf of Maine, Gulf of St. Lawrence, and East Canadian Shelf (Nova Scotia); (2) Arctic: Baffin Bay, Hudson Bay, Davis Strait, Labrador Shelf, East Canadian Shelf (Newfoundland), and Fram Strait (Svalbard); and (3) Northeast Atlantic: Denmark Strait (Iceland) and the Baltic Sea. Regions of secondary contact appear to exist in northern Baffin Bay and the Northwest Atlantic. Possible reasons for this pattern of genetic structure include historical isolation in multiple glacial refugia during the Pleistocene and contemporary barriers to gene flow. Comparison of several potential historical scenarios provided strongest support for isolation of black guillemots in two glacial refugia in the Northwest and Northeast Atlantic, followed by range expansion and secondary contact in the Arctic since recession of the glaciers. Our results suggest that management of black guillemots will require an internationally coordinated approach to conserve the genomic variation within this species.

## Graphical abstract

Identifying distinct genetic populations is important for successful species conservation and management. We used double‐digest restriction‐site associated DNA sequencing (ddRADseq) to look at how 172 black guillemots from different regions in the Arctic and North Atlantic oceans differed genetically. Population genetic analyses identified three genetic clusters: (1) Northwest Atlantic: Gulf of Maine, Gulf of St. Lawrence, and East Canadian Shelf (Nova Scotia); (2) Arctic: Baffin Bay, Hudson Bay, Davis Strait, Labrador Shelf, East Canadian Shelf (Newfoundland), and Fram Strait; and (3) Northeast Atlantic: Denmark Strait and the Baltic Sea. Comparison of four potential historical scenarios provided the strongest support for isolation of black guillemots in two glacial refugia in the Northwest and Northeast Atlantic, followed by range expansion and secondary contact in the Arctic since recession of the glaciers.

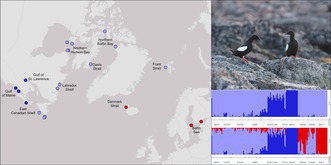

## 1 Introduction

Globally, marine and coastal environments are threatened by pollution, overexploitation, and climate change (Halpern et al. 2007; Brown et al. 2018). Seabirds, as apex predators in these environments, are similarly under a wide range of threats (Dias et al. 2019). In the Arctic, the impacts of climate change are amplified, with warming progressing faster than in the rest of the world (Rantanen et al. 2022). Sea ice extent is rapidly declining, with longer periods between sea ice break‐up and formation becoming more common in recent decades (Parkinson and DiGirolamo 2016; Environment and Climate Change Canada 2023). The rate at which these changes are taking place raises the question of whether marine and coastal ecosystems, and specifically seabirds, will be able to adapt. Quantifying genetic diversity within and among local populations is key to understanding the vulnerability of species to climate change and other pressures (Allendorf et al. 2013). Species with low genetic diversity have a potentially higher risk of extinction, as they have less standing genetic variation for natural selection to act upon (Barrett and Schluter 2007; Allendorf et al. 2013). Population genetic structure has been assessed in several northern seabirds, with direct implications for conservation and management (Lombal et al. 2020).

Black guillemots ( *Cepphus grylle* , hereafter: guillemots, also called pitseolak (ᐱᑦᑎᐅᓛᖅ) or pigeons in some regions covered by this study) are colonial seabirds that breed throughout the Arctic and North Atlantic oceans (Butler et al. 2020). Unlike most other alcids, they breed at low densities along coasts rather than concentrating in a few large colonies. Their sensitivity to changes in coastal habitats at a regional scale makes them useful indicators of ecosystem health (Dehnhard et al. 2023). Guillemots are a culturally important species for many northern Indigenous communities and are legally harvested in Canada, Greenland, Iceland, the Faroe Islands, Russia, and Alaska (USA) (Merkel and Barry 2008). Guillemots are not considered to be globally threatened (BirdLife International 2023), however they are vulnerable to impacts of human activities and are challenging to census accurately due to their crevice‐nesting behavior, the presence of nonbreeding birds at breeding colonies, and their dispersed distribution (Cairns 1979, 1980).

Based on variation in wing length, tarsus length, culmen length and plumage typically six subspecies of black guillemot are generally accepted, but not always well‐defined on the landscape (Storer 1952; Butler et al. 2020, Figure 1). *C. g. mandtii* breeds throughout the high arctic, from northern Yukon and Alaska along the north coast of Russia through to Svalbard; *C. g. ultimus* breeds in the eastern Canadian Arctic, Hudson Bay, Labrador, northern Newfoundland and coastal Greenland; *C. g. arcticus* breeds south of *C. g. mandtii* and *ultimus* in eastern North America, southern Greenland, northern Europe; *C. g. islandicus* breeds around Iceland; *C. g. faeroeensis* is resident to the Faroe Islands; and *C. g. grylle* breeds in the Baltic Sea (Udvardy 1963; Butler et al. 2020).

**FIGURE 1:**
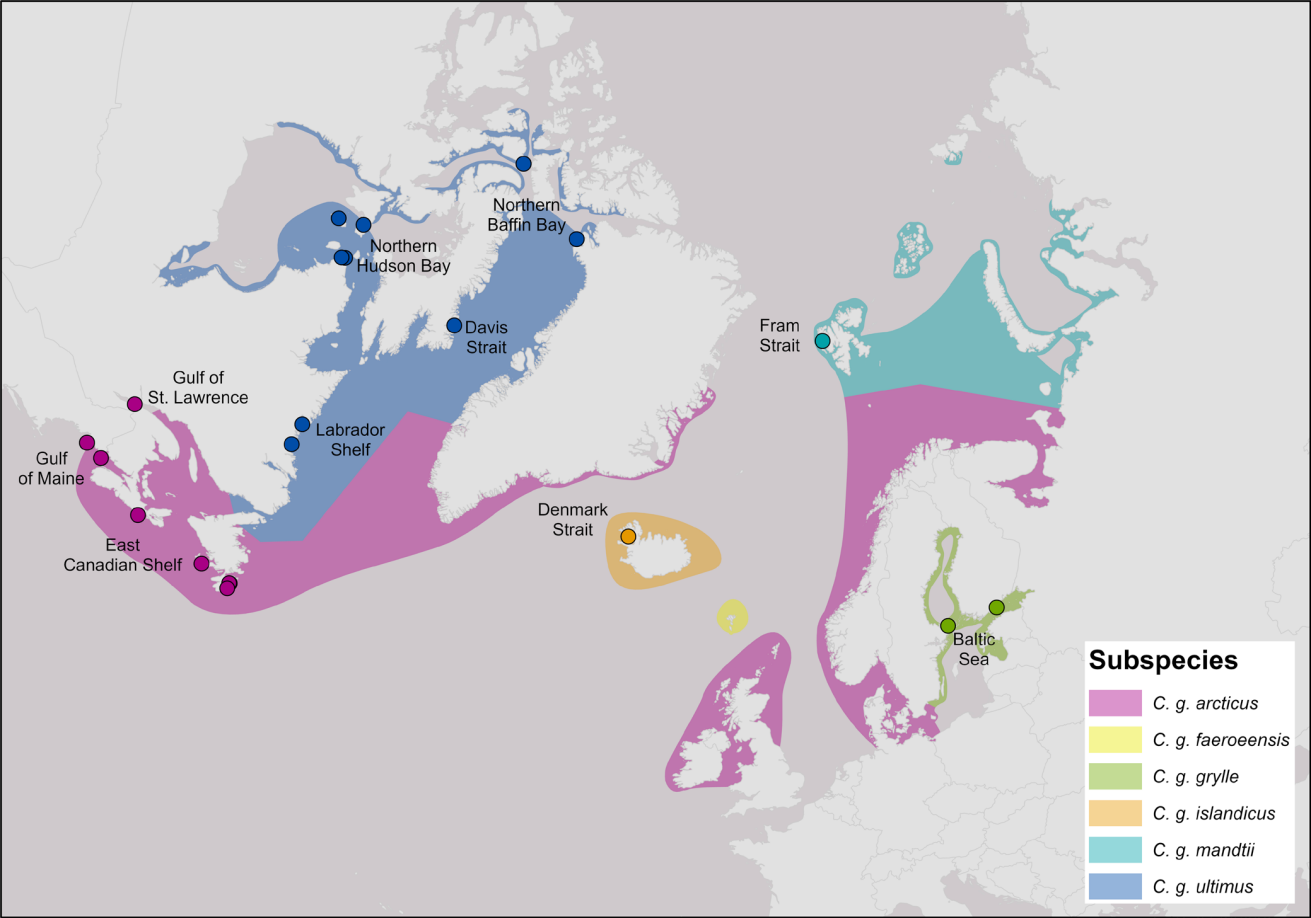
Map of black guillemot distribution within the North Atlantic showing subspecies distributions and locations sampled for ddRADseq analysis. Subspecies distributions are approximate. Sampling locations are color‐coded according to subspecies and sampling regions used for analysis are labeled. Maps were created using ArcGIS Pro (v. 3.3.5, ESRI 2024). Sources: BirdLife International and Handbook of the Birds of the World (2022), ESRI (2022).

Very little is known about population genetic variation in black guillemots, and how this may overlap with the morphological differences between subspecies. Aspects of their behavior, such as high breeding philopatry, inshore foraging, and limited dispersal distances during the winter could contribute to reduced gene flow and potential population genetic differentiation (Nettleship and Birkhead 1985; Friesen 2015). Morphological differences between subspecies could be a result of restricted gene flow. In addition, black guillemots occupy different climate regions: more northern locations experience polar climates, with close pack ice or solid ice cover much of the year, while southern locations experience more temperate climates, either completely ice free or with only open pack ice (Walsh 2008; Environment and Climate Change Canada 2023). However, the extent to which dependency on pack ice exhibited by Arctic guillemots (sometimes referred to as “ice obligates”, Divoky et al. 2021) represents genetic differences versus phenotypic plasticity is unknown.

Using mitochondrial DNA (mtDNA) sampled from seven colonies in the Arctic and Atlantic Oceans, Kidd and Friesen (1998) found that samples from all colonies differed genetically, although genetic divergence did not correlate with either geographic distance or subspecies. Modern genomic techniques such as reduced representation sequencing provide increased resolution of genetic structure, in many cases allowing more robust inferences regarding genetic differentiation among populations than previous assessments based on a limited number of markers (Toews et al. 2016). We conducted a genome‐wide survey of single nucleotide polymorphisms (SNPs) identified by double‐digest restriction‐site associated DNA sequencing (ddRADseq, Peterson et al. 2012) to determine the extent to which black guillemots sampled across a wide geographic span differ at presumptively neutral markers. We tested the hypotheses that (1) regional samples and (2) subspecies of black guillemots are genetically distinct, and (3) that this differentiation developed in part due to historical isolation in glacial refugia.

## 2 Methods

### 2.1 Sample Collection and DNA Extraction

Blood, feather, or tissue samples were collected from 212 black guillemots from 21 breeding locations within their range in the Atlantic and Arctic oceans (Figure 1). All birds sampled were adults or chicks at breeding colonies, except those from Nain, Labrador, which were harvested juveniles. Samples included representation from all subspecies except *C. g. faeroeensis*. Samples are archived at −80°C at Queen's University, Ontario. DNA was purified from samples using a standard proteinase‐K phenol/chloroform extraction and ethanol precipitation (Sambrook et al. 1989). DNA was checked for quality by electrophoresis through agarose, and concentrations were standardized to 20 ng/μL using a Qubit 3.0 fluorometer and a Qubit dsDNA Broad Sensitivity Assay Kit (Invitrogen, Carlsbad, California, USA).

### 2.2 Library Preparation and Quality Filtering

Purified DNA samples were sent to l'Institut de biologie integrative et des systèmes (IBIS) de L'Université Laval, Quebec for ddRADseq library construction. The restriction enzymes *SbfI* and *MspI* were used to cut the DNA into fragments. Fragments were then ligated to two adapters, one of which was a DNA barcode unique to each individual and one that was a common adapter (Davey and Blaxter 2010). Fragments were size‐selected using a BluePippin bioanalyzer (Sage Science), and the resulting RADseq libraries were sequenced either on a HiSeq 2000 using single‐read 100 bp sequencing at the Genome Quebec Innovation Center (McGill University, Montreal, Quebec), or on a HiSeq 2500, using single‐read 100 bp sequencing at The Centre for Applied Genomics (TCAG) at The Hospital for Sick Children, Ontario.

### 2.3 Sequence Processing

The quality of the raw sequence data was assessed using FastQC (v. 2.0.4, Andrews 2010). Adapter content was removed using Trimmomatic (v. 0.40, Bolger et al. 2014). Sequences were demultiplexed, quality filtered, and aligned to the black guillemot reference genome (Feng et al. 2020, GCA_013401065.1; coverage depth = 115×) following the process outlined in Colston‐Nepali et al. 2020 and using STACKS (v. 2.68, Catchen et al. 2013).

Aligned reads were assembled using the *ref_map.pl* pipeline in STACKS. The STACKS program *populations* was used to filter for a minimum minor allele frequency of 1% and a maximum observed heterozygosity of 75%. Data analysis was restricted to the first SNP per locus. Loci were further filtered in VCFtools (v. 0.1.16, Danecek et al. 2011) for a minimum depth of 5, a maximum depth of 120, and a maximum of 20% missing data. Individuals with more than 20% missing data were removed. Loci were screened for deviations from Hardy–Weinberg proportions within sampling regions (Pearman et al. 2021).

Samples were sequenced in three batches due to samples being collected over multiple field seasons. Several sampling regions were represented in two or more batches, to reduce potential batch effects. In addition, several samples were repeated in both batches one and two, and batches one and three to correct for potential batch effects including differences in sequencing platforms. Discordance between replicates was tested using both the file comparison option –diff‐indv‐discordance in VCFtools and a principal component analysis (PCA) using the R (v. 4.3.1, R Core Team 2022) packages adegenet (Jombart and Ahmed 2011) and ade4 (Dray and Dufour 2007). Average discordance between batches one and two was 1% and average discordance between batches one and three was 3.3%. This, combined with the results from the PCA, suggests that discordance between batches was minimal.

After filtering and removal of duplicate samples, 172 individuals and 3834 SNPs remained (dataset 1). The data were further filtered in PLINK (v. 1.9, Purcell et al. 2007) to remove SNPs with a correlation coefficient of 0.2 or higher (dataset 2; 172 individuals, 3724 SNPs). For demographic analyses, data were filtered similar to dataset 1 but without filtering for minor allele frequency (dataset 3; 172 individuals, 6444 SNPs). Information on filtering parameters and number of individuals and SNPs for each step of filtering is given in Table S1.

### 2.4 Population Genetic Structure Analyses

As outlined in our hypothesis, samples were grouped and analyzed by oceanographic region (referred to as regional populations). PGDSpider (v. 3.0.0, Lischer and Excoffier 2012) and PLINK were used to convert VCF files to appropriate formats for subsequent analysis. To test for population genetic structure, global *F*_ST_ was calculated using Weir and Cockerham's weighted *F*_ST_ (Weir and Cockerham 1984), and *F*_ST_ between sampling regions was calculated with an analysis of molecular variance (AMOVA) in Genodive (v. 3.06, Meirmans and van Tienderen 2004). *F*_ST_ estimates were tested for significance using 10,000 permutations. *p*‐values for pairwise comparisons were adjusted for multiple comparisons using Benjamini‐Yekutieli corrections (Benjamini and Yekutieli 2001; Narum 2006).

The program STRUCTURE (v. 2.3.4, Pritchard et al. 2000, 2010), run in StrAuto (v. 1.0, Chhatre and Emerson 2017) was also used to test for population genetic structure (dataset 2). Each run used a burn‐in of 100,000 followed by 1,000,000 MCMC iterations, using the admixture model with correlated allele frequencies and without sampling region as prior information. To assess the most probable number of genetic clusters in our data, each value of *K* from one to eight was run five times and assessed with both the parsimony indicator implemented in KFinder (v. 2.1, Wang 2019) and delta *K* (Δ*K*) using STRUCTURE HARVESTER (v. 0.7, Evanno et al. 2005; Earl and vonHoldt 2012). Barplots were created with the R package pophelper (Francis 2016). Population genetic structure was also assessed using a PCA of regional populations, as well as a discriminant analysis of principal components (DAPC) of regional populations. The DAPC was performed using the R packages adegenet and ade4. Missing data were replaced with mean allele frequencies (Jombart et al. 2010). *K*‐means clustering and Bayesian Information Criterion (BIC) model selection were used to identify the optimal value of *K*, which was determined to be one. Sampling regions were therefore used to group samples for the DAPC. Cross‐validation was used to identify how many PCs should be retained in the DAPC, with 1000 replicates.

A Mantel test (Mantel 1967) was used to test for a correlation between genetic distance and geographic distance between sampling regions. We also performed two additional Mantel tests, excluding those sampling regions that were differentiated in prior analyses to assess how these impacted the relationship between genetic distance and geographic distance. Pairwise *F*_ST_ values were converted to Slatkin's linearized *F*_ST_ as the measure of genetic distance. Geographic distances were calculated as the least cost path distances (over water only; Natural Earth 2009) between sampling regions in ArcGIS (v. 10.8.1, ESRI 2020). Where multiple sampling locations were grouped into one regional population, a geographic midpoint was calculated. Distances were then log‐transformed (Rousset 1997). The Mantel tests were executed with the R package ecodist (100,000 permutations; Goslee and Urban 2007). Regions with less than ten samples were excluded from these analyses.

### 2.5 Demographic Modeling

Four evolutionary models were compared using *fastsimcoal2* (v. 2.8, dataset 3; Excoffier et al. 2013, 2021, 2023). *Fastsimcoal2* is a maximum likelihood‐based method which uses coalescent simulations to model complex demographic scenarios from the site frequency spectrum (SFS; Excoffier et al. 2013). For simplicity, alternative topologies were used to represent different isolation and range expansion scenarios; samples from Maine, New Brunswick and Nova Scotia were grouped as “Northwest Atlantic”; samples from the Baltic Sea and Denmark Strait were grouped together as “Northeast Atlantic”, and all other samples were grouped together as “Arctic”. Model 1 represented survival of black guillemots in a single refugium in the western Atlantic, with expansion north and eastward starting ~8000 years ago (ya) until the present (similar to the postulated evolutionary history of razorbills ( *Alca torda* ), Moum and Árnason 2001). Model 2 represented survival of black guillemots in a single refugium in the Northeast Atlantic, with expansion north and westward starting ~8000 ya until the present. Model 3 represented divergence in two Wisconsin refugia (one in the Northwest Atlantic; one in the Northeast Atlantic) prior to ~8000 ya, with the Arctic population formed by expansion and contemporary introgression of the refugial populations ~4000 ya (similar to common murres ( *Uria aalge* ), Morris‐Pocock et al. 2008). And Model 4 represented divergence of guillemots in two Illinoisian refugia prior to ~12,000 years ago (Northwest Atlantic and ancestral Arctic), divergence of the ancestral Arctic population into contemporary Arctic and Northeast Atlantic during the Wisconsin glaciation (~8000 ya), with subsequent gene flow among all three populations (Kidd and Friesen 1998). Divergence times were fixed in all scenarios, population sizes were held constant and the contributions of the ancestral populations in the Arctic (Model 3) was set at 50/50 since our purpose was only to test models of ancestral vicariance and range expansion. The 2D folded site frequency spectrum (SFS) was constructed using *easySFS* (https://github.com/isaacovercast/easySFS; Gutenkunst et al. 2009). *Fastsimcoal2* was run using 10,000 coalescent simulations and 20 optimization cycles for parameter estimation. 100 independent runs were performed for each demographic model and results for the estimation with the highest likelihood are reported. Model fit was determined using Akaike Information Criterion (AIC) values. A generation time of 8 years (Bird et al. 2020; Harkness et al. 2024) and mutation rate of 10e‐8 (Kersten et al. 2023) were assumed.

## 3 Results

### 3.1 Population Genetic Analysis

**FIGURE 2:**
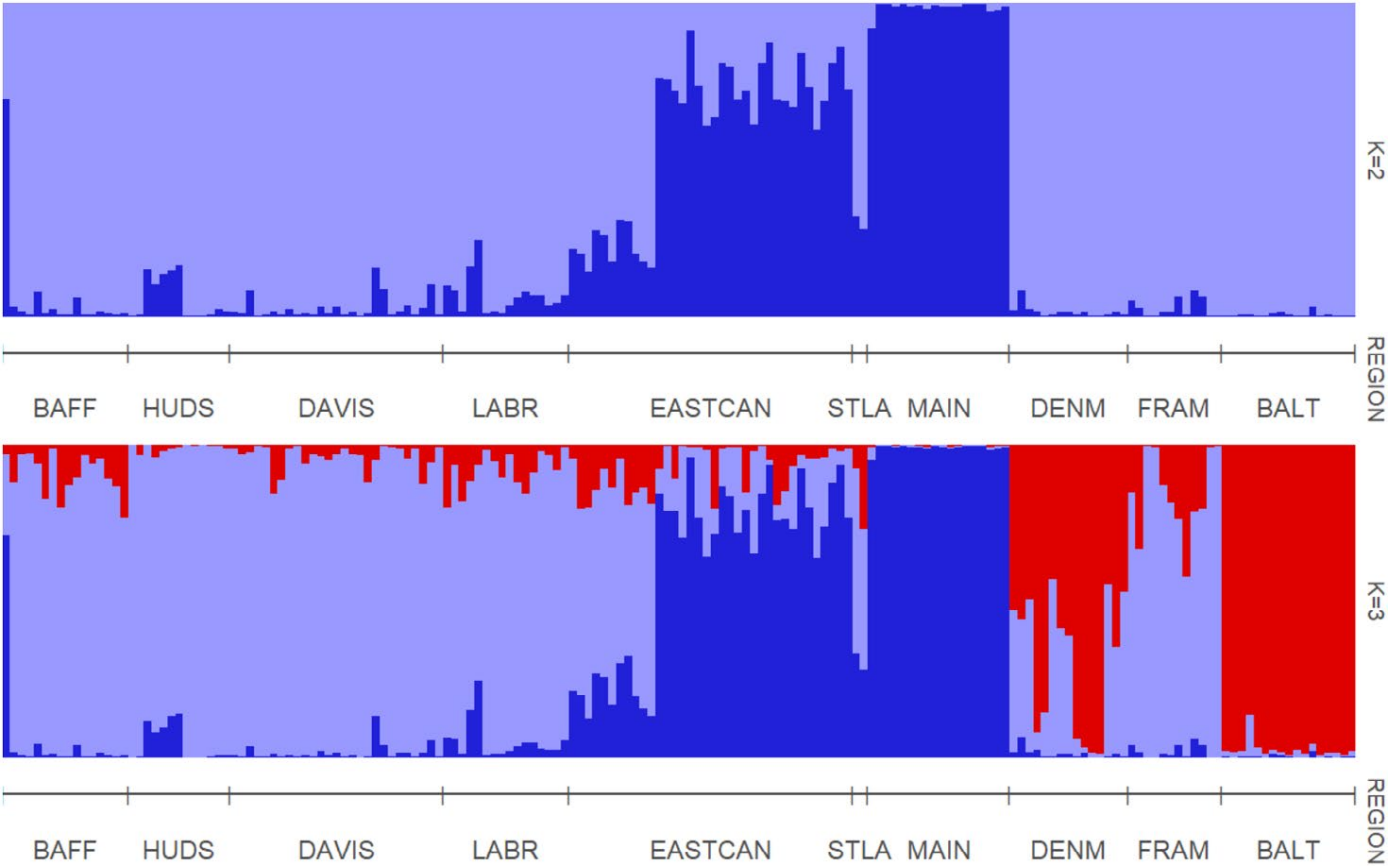
Probabilities of assignment of individual guillemots to different genetic populations from STRUCTURE analyses. Results are shown for the most likely value of *K* as identified by STRUCTURE HARVESTER (top) and the parsimony indicator in KFinder (bottom). See Table 1 for population abbreviations.

The estimate of global population genetic structure (*F*_ST_) was 0.028 and estimates of *F*_ST_ between pairs of regional samples were significant for most comparisons (Table 2). Results from STRUCTURE differed slightly depending on the method for estimating best *K*. The parsimony method in KFinder indicated that our black guillemot samples most likely comprise three genetic groups (*K* = 3, Figure 2). Guillemots sampled from the Gulf of Maine, Gulf of St. Lawrence, and part of the East Canadian Shelf had a high probability of assignment primarily to one genetic population; guillemots sampled from Baffin Bay, Hudson Bay, Davis Strait, the Labrador Shelf, part of the East Canadian Shelf and Fram Strait assigned primarily to a second genetic population; while those sampled from Denmark Strait and the Baltic Sea assigned primarily to a third genetic population, with varying probabilities. Results from delta *K* (Δ*K*) in STRUCTURE HARVESTER (Evanno et al. 2005; Earl and vonHoldt 2012) suggested that the optimal value for *K* was two (*K* = 2, Figure 2). The pattern of differentiation was similar to that seen at *K* = 3, with the exception of guillemots sampled from Denmark Strait and the Baltic Sea grouping with the Arctic population. Higher values of *K* start to differentiate between guillemot samples from Denmark Strait and the Baltic Sea (Figure S1).

**FIGURE 3:**
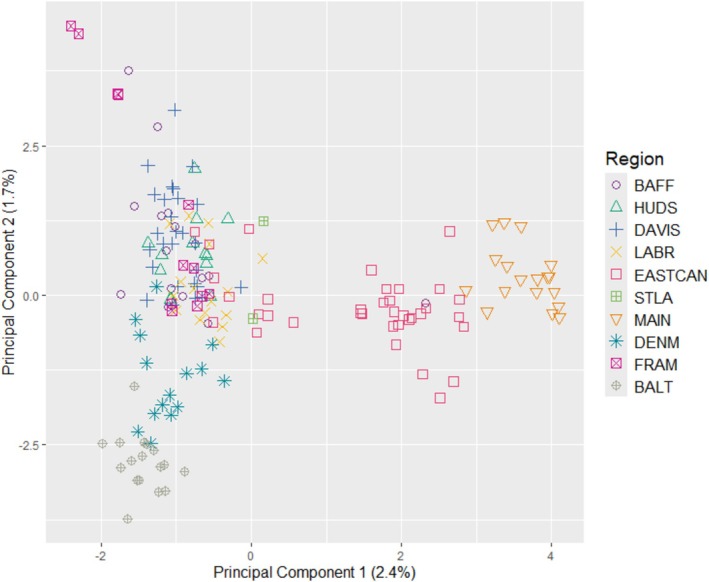
The first two principal components of a principal components analysis (PCA) of black guillemot samples using 3834 SNPs. The first two principal components explain 4.1% of the variance. See Table 1 for region abbreviations.

**FIGURE 4:**
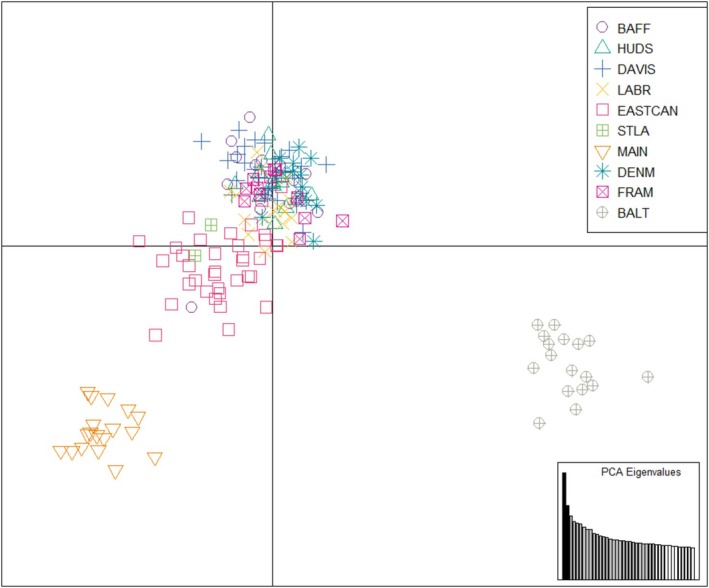
A discriminant analysis of principal components (DAPC) of black guillemot samples using 3834 SNPs. PCA eigenvalues are shown in the bottom right, with retained PCs in gray and plotted PCs in black. See Table 1 for region abbreviations.

**FIGURE 5:**
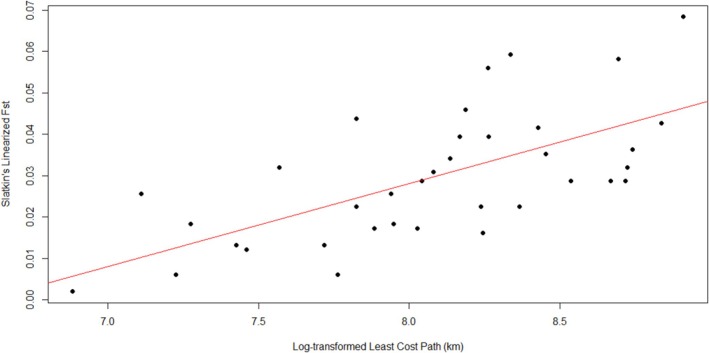
Results of a Mantel test using Slatkin's linearized *F*_ST_ and log‐transformed least cost path distance between regional populations to test for a correlation between genetic distance and geographic distance in black guillemot samples. The red line indicates the significant positive relationship between genetic and geographic distance (*r* = 0.66, *p* < 0.01). (Analysis was conducted using *F*_ST_ values calculated for regions with 10 samples or more).

Results from the PCA showed some differentiation between regional samples (Figure 3), with the first two principal components explaining 4.1% of the variance. Guillemots from the Gulf of Maine, Gulf of St. Lawrence, and part of the East Canadian Shelf were spread along the axis of the first principal component, separate from other regional samples. Guillemots from the Baltic Sea clustered together, overlapping with some guillemots from the Denmark Strait at one extreme of the second principal component. Guillemots from Baffin Bay, Hudson Bay, Davis Strait, the Labrador Shelf, Fram Strait, and several individuals from the East Canadian Shelf and Denmark Strait clustered closely together. Results from the DAPC were similar (Figure 4), separating guillemots from both the Gulf of Maine and the Baltic Sea into distinct clusters along the first axis. Guillemots from the Gulf of St. Lawrence and some from the East Canadian Shelf clustered together, showing some differentiation from other regions along the second axis. Remaining regions were again clustered closely together. The Mantel test with all sampling regions showed a significant positive relationship between genetic and geographic distances (*r* = 0.66, *p* < 0.01, Figure 5). A second test excluding guillemot samples from Denmark Strait and the Baltic Sea also showed a significant positive relationship between genetic and geographic distances (*r* = 0.67, *p* < 0.01), as did a third test excluding guillemot samples from the Gulf of Maine and the East Canadian Shelf (*r* = 0.75, *p* < 0.01).

### 3.2 Demographic History

**FIGURE 6:**
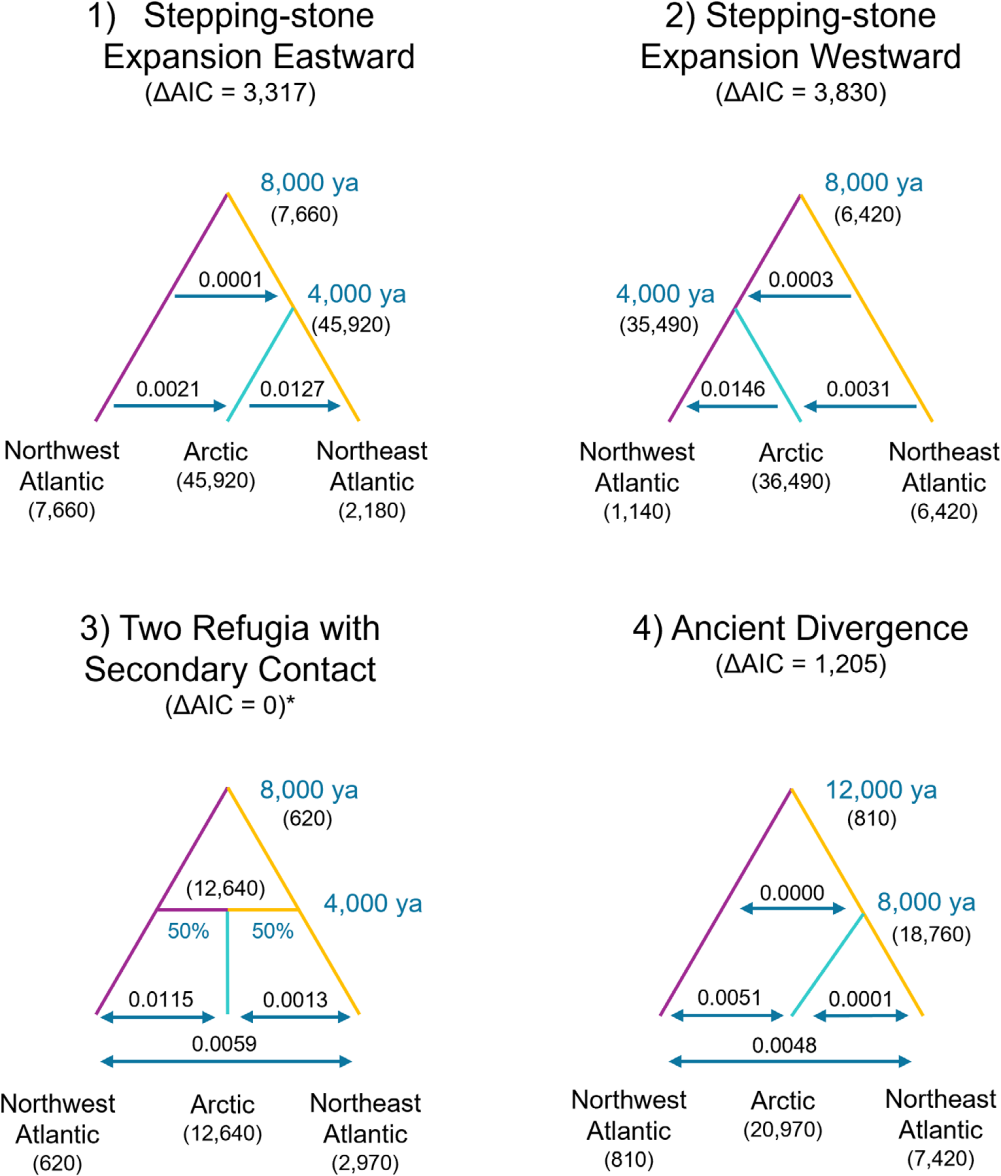
Results of coalescent modeling of four historical scenarios for black guillemots using *fastsimcoal2*. Parameters in blue were provided to the program; estimates in black are results from most likely simulations. Arrows indicate direction of migration going forward in time; numbers above arrows indicate migration rates (proportion of population). Numbers in parentheses are estimated population sizes.

The best supported model in *fastsimcoal28* was Model 3 (the one with the lowest AIC), where guillemots diverged in two Wisconsin refugia (Northwest Atlantic and Northeast Atlantic) until ~8000 ya, with the Arctic population formed by secondary contact of the refugial populations ~4000 ya (Figure 6). No other models had a ΔAIC lower than 10, suggesting low support for alternative scenarios of demographic history.

## 4 Discussion

### 4.1 Population Genetic Structure

We conducted a genome‐wide survey of SNPs identified by ddRADseq to determine the extent to which black guillemots sampled across a wide geographic span differ at neutral markers. We tested the hypotheses that regional populations and subspecies of black guillemots are genetically distinct, and that this differentiation was in part due to historical isolation in glacial refugia.

In support of our first hypothesis, we found significant genetic structure among regional populations of black guillemots. The majority of pairwise F_ST_ estimates were statistically significant (Table 2), and population structure was evident in the results from STRUCTURE (Figure 2), PCA (Figure 3), and DAPC (Figure 4). Birds sampled from the Gulf of Maine, Gulf of St. Lawrence, and part of the East Canadian Shelf (Nova Scotia) were differentiated from those elsewhere (hereafter: Northwest Atlantic population). The remaining East Canadian Shelf samples (Newfoundland) showed higher potential assignment to the same genetic population as birds in Baffin Bay, Hudson Bay, Davis Strait, Labrador Shelf, and Fram Strait (hereafter: Arctic population). Birds from the Baltic Sea were also differentiated from those elsewhere, showing little to no probability of assignment to either of the other genetic populations. In results from STRUCTURE and PCA, birds from Denmark Strait showed high potential assignment to the same genetic population as birds from the Baltic Sea (hereafter: Northeast Atlantic population), with some potential assignment to the Arctic population. This relationship was not present in the DAPC, with birds from the Denmark Strait showing high potential assignment to the Arctic population. Birds from Fram Strait were more genetically similar to the Arctic population than the Northeast Atlantic population, despite them being similar distances apart over water. Although birds from Hudson Bay did not form their own genetic population in the STRUCTURE, PCA, and DAPC analyses, F_ST_ values indicated that they were differentiated from birds in the Gulf of Maine, East Canadian Shelf, Denmark Strait, Fram Strait, and Baltic Sea (Table 2). We found a positive correlation between genetic and geographic distance between regional populations (Figure 5). Excluding birds from the Northeast Atlantic did not impact the strength of the correlation. However, the correlation was stronger when birds from the Northwest Atlantic were excluded.

Our results are in concordance with previous research by Kidd and Friesen (1998), who found strong population genetic structure in black guillemots in the mitochondrial control region. Both studies detected differentiation between regional populations. Similar levels of population genetic structure were found in pigeon guillemots ( *Cepphus columba* , Kidd and Friesen 1998; Harkness et al. 2024), which contrasts with weak or absent genetic population differentiation in many but not all high latitude Northern Hemisphere seabird species (Friesen 2015; Lombal et al. 2020). Population genetic structure in black guillemots could result from any or all of at least three main factors: historical fragmentation, restrictions to contemporary gene flow, and local adaptation.

#### 4.1.1 Historical Fragmentation

The pattern of differentiation that we see in black guillemots could, at least partially, be explained by isolation of guillemots in two or more glacial refugia during the Pleistocene. In the Arctic, the much colder climate and coverage by ice sheets at the Last Glacial Maximum (LGM) would generally have pushed species to lower latitudes (Hewitt 2004). However, several regions remained ice‐free during this time and could have acted as glacial refugia for Arctic vertebrates. In the North Atlantic and Arctic these refugia included the Canadian Arctic Archipelago, western Greenland, Newfoundland Bank, Spitsbergen Bank, Iceland and western Europe (Holder et al. 1999; Sonsthagen et al. 2011). Phylogeographic studies of other alcid species have identified Pleistocene refugia in both North America and Europe. Common murres from the Atlantic show east–west substructure suggesting they were isolated in two Pleistocene refugia, followed by expansion and secondary contact (Morris‐Pocock et al. 2008). Razorbills likely originated from a north‐western Atlantic refugial population with subsequent expansions east and north (Moum and Árnason 2001). Atlantic puffin ( *Fratercula arctica* ) genomic structure and demographic history suggest that a high Arctic population may have been isolated in a refugium during the LGM, followed by secondary contact and colonization of the North Atlantic by more southern populations post‐LGM (Kersten et al. 2021, 2023). Seabird species from other families (common eiders ( *Somateria mollissima* ), Sonsthagen et al. 2011; white‐headed gulls (*Larus* spp.), Sonsthagen et al. 2012; black‐legged kittiwakes ( *Rissa tridactyla* ), Sauve et al. 2019) also show genetic evidence of isolation in multiple Arctic and north Atlantic refugia. Black guillemots were estimated by Kidd and Friesen (1998) to have undergone three periods of diversification: divergence of guillemots in Maine and the Chukchi Sea from other populations approximately 0.5 millions of years before present (M.Y.B.P.); divergence of Maine guillemots from those in the Chukchi Sea approximately 0.25 M.Y.B.P.; and radiation of guillemots in the North Atlantic Ocean and Norwegian Sea approximately 0.06–0.08 M.Y.B.P (during the Wisconsin glaciation). Kidd and Friesen (1998) concluded that birds from the Gulf of Maine and Baltic Sea had remained isolated since retreat of the glaciers, while gene flow appeared to have resumed among birds from the Chukchi Sea, Denmark Strait, Norwegian Sea, and Canadian Arctic.

Our demographic history results support the proposal that black guillemots previously diverged in two Wisconsin refugia (one in the Northwest Atlantic and one in the Northeast Atlantic) prior to ~8000 ya. The Arctic population was then formed by expansion, secondary contact and subsequent contemporary interbreeding of the refugial populations ~4000 ya (Figure 6). This is further supported by mixed assignment probabilities observed in Arctic regions, indicating a possible zone of secondary contact and introgression in Baffin Bay and surrounds. A previous theory suggested that birds in the Baltic Sea may have arrived in these areas postglaciation (Brown 1985) and differentiated from those elsewhere due to contemporary barriers to gene flow (Lombal et al. 2020), however, this scenario was not supported by our model selection. Iceland has been identified as the location of a possible population genetic or phylogeographic break for seabirds (Friesen et al. 2007), which is supported by our results.

### 4.2 Contemporary Gene Flow

Previously, guillemots were believed to have strong natal philopatry (Nettleship and Birkhead 1985), although more recent banding studies have found that natal dispersal in black guillemots may be relatively high, at least within regions (Frederiksen and Petersen 2000), and past estimates of philopatry in seabirds may be exaggerated (Coulson 2016). Young guillemots (and other auk species) disperse farther than adults, especially in their first winter (Brown 1985). Dispersal of young guillemots could, in part, contribute to gene flow within oceanographic regions and the pattern of population genetic structure we found in black guillemots.

Given that most black guillemots stay close to their breeding colonies during the summer, overlap in distributions during the nonbreeding season could result in the exchange of migrants between colonies, maintaining gene flow among regions (Friesen et al. 2007). Distributions of guillemots in the Arctic are largely influenced by the formation and melt of sea ice, with the northern limit of their nonbreeding distribution defined by the availability of open water near ice, either in polynyas or at the edges of pack ice (Bradstreet 1979; Divoky et al. 2016). In ice‐free areas, guillemots remain close to their breeding colonies during the nonbreeding season (Nettleship and Birkhead 1985). Distributions are also associated with prey availability and areas of high productivity (Cairns 1987; Divoky et al. 2016). Using global location sensors (GLS) and stable isotopes, Baak et al. (2021) found that breeding guillemots from Country Island, eastern Nova Scotia, overwintered on the Scotian Shelf, in the Gulf of St. Lawrence, Bay of Fundy or Gulf of Maine, whereas guillemots from Kent Island, New Brunswick, remained in the Bay of Fundy or Gulf of Maine. This overlap in nonbreeding distribution could, in part, explain the potential admixture we found between guillemots in the Northwest Atlantic. There are no other published telemetry data available for black guillemots in the nonbreeding season within our study area; however, guillemots are common in Hudson Strait, Hudson Bay, and Davis Strait during the winter, and are also found in polynyas in the Canadian high Arctic and along the ice edge at Lancaster Sound (Butler et al. 2020). European guillemots do not typically move far from their breeding colonies, with some exceptions (Butler et al. 2020). Our demographic modeling results indicate that there is very little migration between genetic clusters.

Genetic differentiation among regional populations showed a strong positive correlation with geographic distance (measured over water). This correlation can result when a species' dispersal is constrained by distance so that gene flow is most likely to occur between neighboring colonies (“isolation by distance” following a “stepping stone” model of dispersal; Wright 1943; Kimura and Weiss 1964; Hutchison and Templeton 1999). As a result, guillemots that breed closer together geographically would tend to be more genetically similar. This supports our current understanding of dispersal in guillemots, as they are unlikely to fly over large masses of land or open water, preferring to remain close to shorelines, and do not make long‐distance seasonal migrations, potentially minimizing the opportunity for gene flow. Evidence for isolation by distance has been found in many seabird species, although in many cases geographic distance has been found to correlate only weakly with population genetic structure (e.g., black‐legged kittiwake, Friesen et al. 2007). Kidd and Friesen (1998) found no evidence of a correlation between genetic and geographic distance (measured both linearly and along the shoreline between colonies), however our study used a higher number of sampling locations and a different geographic sampling distribution. Genetic differences between sampling locations correlated with geographic distance in pigeon guillemots (Harkness et al. 2024). Distance is likely a key factor in the maintenance of genetic differentiation between black guillemot breeding colonies, along with historical barriers to gene flow, and distribution and foraging ecology during the nonbreeding season.

### 4.3 Local Adaptation

Genetic differences between regional populations could reflect local adaptations. Within our sampling distribution, black guillemots experience a range of climates and sea ice cover. Arctic regions experience polar climates and summer sea ice, whereas North Atlantic regions experience more temperate climates and little to no sea ice (Walsh 2008; Environment and Climate Change Canada 2023). Black guillemots from the Chukchi Sea have shown reduced fitness from changes in prey availability due to decreased extent of sea ice and increased sea surface temperature (Divoky et al. 2015). In this region, movement and distribution during the nonbreeding season is also closely tied to the presence of sea ice (Divoky et al. 2016). Our results suggest that birds sampled from regions that experience winter sea ice (Labrador Strait, Hudson Bay, Davis Strait, Baffin Bay, and Fram Strait) are genetically differentiated from birds sampled from regions that experience little to no sea ice (Baltic Sea, Denmark Strait, East Canadian Shelf, Gulf of St. Lawrence, and Gulf of Maine), with the notable exception of Newfoundland birds, which are more genetically similar to the northern, ice‐associated populations. Given that we assayed presumably neutral genetic variation, additional work is needed to determine if genetic differentiation between sampling regions is associated with adaptation to different environments.

### 4.4 Taxonomic Implications and Future Directions

Subspecies of black guillemots were previously designated based on variation in morphology, plumage, and distribution (Storer 1952; Butler et al. 2020; Table 1, Figure 1). Contrary to our second hypothesis, differentiation at neutral markers did not fully support current subspecies delineations. According to Butler et al. (2020), both *C. g. grylle* and *C. g. mandtii* are relatively easily identifiable by size and plumage, while *C. g. islandandicus* and *C. g. faeroeensis* are more difficult, if not impossible to differentiate by physical characteristics. In addition, *C. g. arcticus* and *C. g. ultimus* are difficult to partition into separate subspecies due to clinal variation. Our results show that birds from Denmark Strait (*C. g. islandicus*) are genetically similar to those in the Baltic Sea (*C. g. grylle*). However, we do see some differentiation between these two regions at higher values of *K* (Figure S1) and in a PCA. Our study did not include samples from the Faroe Islands, so we are not able to conclude whether *C. g. faeroeensis* is genetically different from other nearby subspecies. Birds from Gulf of Maine, Gulf of St. Lawrence and East Canadian Shelf (Nova Scotia, *C. g. arcticus*) were genetically similar to each other, although introgression from Arctic guillemots may occur along the eastern coast of Canada. Genetic similarity among birds from the East Canadian Shelf (Newfoundland, *C. g. arcticus*), Labrador Shelf, Davis Strait, Hudson Bay, Baffin Bay (*C. g. ultimus*), and Fram Strait (*C. g. mandtii*) does not support the existence of *C. g. ultimus* as a distinct subspecies. This is concordant with some species descriptions, which do not separate *C. g. ultimus* from *C. g. mandtii* (Butler et al. 2020). More samples from *C. g. mandtii* are required to determine if this genetic similarity exists throughout their range.

**TABLE 1:** Subspecies, oceanographic regions, region abbreviations, sampling locations and sample size (*n*) of black guillemots included in analysis of ddRADseq data.

Subspecies	Oceanographic region	Abbreviation	Sampling locations	Dataset 1 and 2	Dataset 3
				*n*	*n*
*C. g. ultimus*	Northern Baffin Bay	BAFF	Prince Leopold Island, Canada	7	10
			Iganak, Greenland	9	9
	Northern Hudson Bay	HUDS	Coats Island, Canada	3	3
			Digges Island, Canada	2	3
			East Bay Island, Canada	5	5
			Green Island, Canada	3	3
	Davis Strait	DAVIS	Qikiqtarjuaq, Canada	27	27
	Labrador Shelf	LABR	Nain, Canada	15	15
			Makkovik, Canada	1	1
*C. g. arcticus*	East Canadian Shelf	EASTCAN	Little Bell Island, Canada	2	3
			Kellys Island, Canada	3	3
			Gull Island, Canada	1	1
			Middle Lawn Island, Canada	5	5
			Country Island, Canada	25	25
	Gulf of St. Lawrence	STLA	Long Pelerin Island, Canada	2	2
	Gulf of Maine	MAIN	Kent Island, Canada	10	11
			Great Duck Island, United States of America	8	8
*C. g. islandicus*	Denmark Strait	DENM	Flatey Island, Iceland	15	15
*C. g. mandtii*	Fram Strait	FRAM	Kongsfjorden, Norway	12	12
*C. g. grylle*	Baltic Sea	BALT	Signildskär, Finland	9	9
			Söderskär, Finland	8	8
Total				172	178

**TABLE 2:** Estimates of *F*_ST_ for pairwise comparisons of black guillemot populations (below diagonal) and associated *p*‐values (above diagonal).

	Baffin Bay	Hudson Bay	Davis Strait	Labrador Shelf	East Canadian Shelf	Gulf of Maine	Denmark Strait	Fram Strait	Baltic Sea
Northern Baffin Bay	—	< 1e‐5	0.221	< 1e‐5	< 1e‐5	< 1e‐5	< 1e‐5	0.004	< 1e‐5
Northern Hudson Bay	**0.017**	—	< 1e‐5	< 1e‐5	< 1e‐5	< 1e‐5	< 1e‐5	< 1e‐5	< 1e‐5
Davis Strait	0.002	**0.013**	—	< 1e‐5	< 1e‐5	< 1e‐5	< 1e‐5	< 1e‐5	< 1e‐5
Labrador Shelf	**0.006**	**0.018**	**0.006**	—	< 1e‐5	< 1e‐5	< 1e‐5	< 1e‐5	< 1e‐5
East Canadian Shelf	**0.016**	**0.028**	**0.018**	**0.012**	—	< 1e‐5	< 1e‐5	< 1e‐5	< 1e‐5
Gulf of Maine	**0.040**	**0.053**	**0.044**	**0.042**	**0.025**	—	< 1e‐5	< 1e‐5	< 1e‐5
Denmark Strait	**0.022**	**0.038**	**0.025**	**0.022**	**0.030**	**0.056**	—	< 1e‐5	< 1e‐5
Fram Strait	**0.013**	**0.034**	**0.017**	**0.022**	**0.028**	**0.055**	**0.031**	—	< 1e‐5
Baltic Sea	**0.028**	**0.041**	**0.031**	**0.028**	**0.035**	**0.064**	**0.033**	**0.038**	—

*Note:* Numbers in bold indicate *p* < 0.01, after adjusting for multiple comparisons. See Table 1 for breakdown of regions and sampling locations (only regions with 10 or more samples were included in this analysis).

Based on our results, we suggest that black guillemots should be managed as at least three genetically differentiated groups: (1) Northwest Atlantic: Gulf of Maine, Gulf of St. Lawrence, and East Canadian Shelf (Nova Scotia); (2) Arctic: Baffin Bay, Hudson Bay, Davis Strait, Labrador Shelf, East Canadian Shelf (Newfoundland), and Fram Strait; and (3) Northeast Atlantic: Denmark Strait and the Baltic Sea. These populations span multiple countries, meaning an internationally coordinated approach will be required to conserve the genomic variation within this species. The use of ddRADseq to sequence thousands of markers allowed us to examine population structure at a higher resolution than was done previously. In addition, the use of a reference genome in combination with RADseq resulted in improved quality of population genetic analyses (Andrews and Luikart 2014). For consideration as a next step, whole‐genome sequencing could be used to explore patterns of adaptive variation and to link genes to phenotypes and environmental variables (Toews et al. 2016). This approach would improve our understanding of how black guillemots might be genetically adapted to local climates and other environmental factors. In particular, investigating the relationship between climate variables (such as increasing temperatures, sea ice loss) and genetic adaptations in black guillemots will be increasingly important for predicting how this species will respond to climate change, including an assessment of their vulnerability to emerging threats and pathogens. Our sample distribution spanned most of the range of black guillemots in the Atlantic and Arctic; however, important sampling gaps exist in the Pacific‐Arctic and Northeast Atlantic region. Future work including samples from the Russian Arctic and Europe will allow more general conclusions about population genetic structure in black guillemots and the overall vulnerability of the species.

## Author Contributions

**Bronwyn A. S. Harkness:** conceptualization (supporting), data curation (supporting), formal analysis (equal), funding acquisition (supporting), investigation (lead), methodology (lead), project administration (supporting), validation (lead), visualization (equal), writing – original draft (lead), writing – review and editing (lead). **Lila Colston‐Nepali:** data curation (supporting), formal analysis (equal), methodology (supporting), validation (supporting), visualization (equal), writing – review and editing (supporting). **Gregory J. Robertson:** conceptualization (supporting), data curation (supporting), funding acquisition (supporting), investigation (supporting), resources (supporting), writing – review and editing (supporting). **Jennifer F. Provencher:** data curation (supporting), investigation (supporting), resources (supporting), writing – review and editing (supporting). **Christopher K. Boccia:** formal analysis (equal), methodology (equal), validation (supporting), writing – review and editing (supporting). **Vicki L. Friesen:** conceptualization (lead), data curation (lead), formal analysis (equal), funding acquisition (lead), investigation (supporting), methodology (supporting), project administration (lead), resources (lead), supervision (lead), writing – review and editing (supporting).

## Funding

This work was supported by Mitacs; Environment and Climate Change Canada; Queen's University; Natural Sciences and Engineering Research Council of Canada (STPGP/493789‐2016).

## Conflicts of Interest

The authors declare no conflicts of interest.

## Supporting information


**Figure S1:** Probabilities of assignment of individual guillemots to different genetic populations from STRUCTURE analyses for values of *K* from 2 to 8. See Table 1 for population abbreviations.

**Table S1:** Information on parameters used for each step of data filtering and the number of SNPs and Individuals that remained following each step. ‘x’ indicates that this parameter was not used for the specified dataset.

## Data Availability

Raw reads from ddRADseq have been deposited to the NCBI Sequence Read Archive (SRA): PRJNA1240337. All data are publicly available as of the date of publication, apart from samples from Nain and Makkovik, Canada which are available upon request at the discretion of the Nunatsiavut Government. Requests for information and data should be directed to Bronwyn. A. S. Harkness (bronwyn.harkness@gmail.com).
