# Supplementary material for: Population Genomic Structure and Demographic History of Black Guillemots Breeding Across the North Atlantic
Source: Ecol Evol. 2026 Feb 24;16(2):e73126. doi: 10.1002/ece3.73126 (PMC12930286; doi:10.1002/ece3.73126)
Supplement: Supplementary file 1 — Figure S1: Probabilities of assignment of individual guillemots to different genetic populations from STRUCTURE analyses for values of K from 2 to 8. See Table 1 for population abbreviations. [file ECE3-16-e73126-s002.docx]

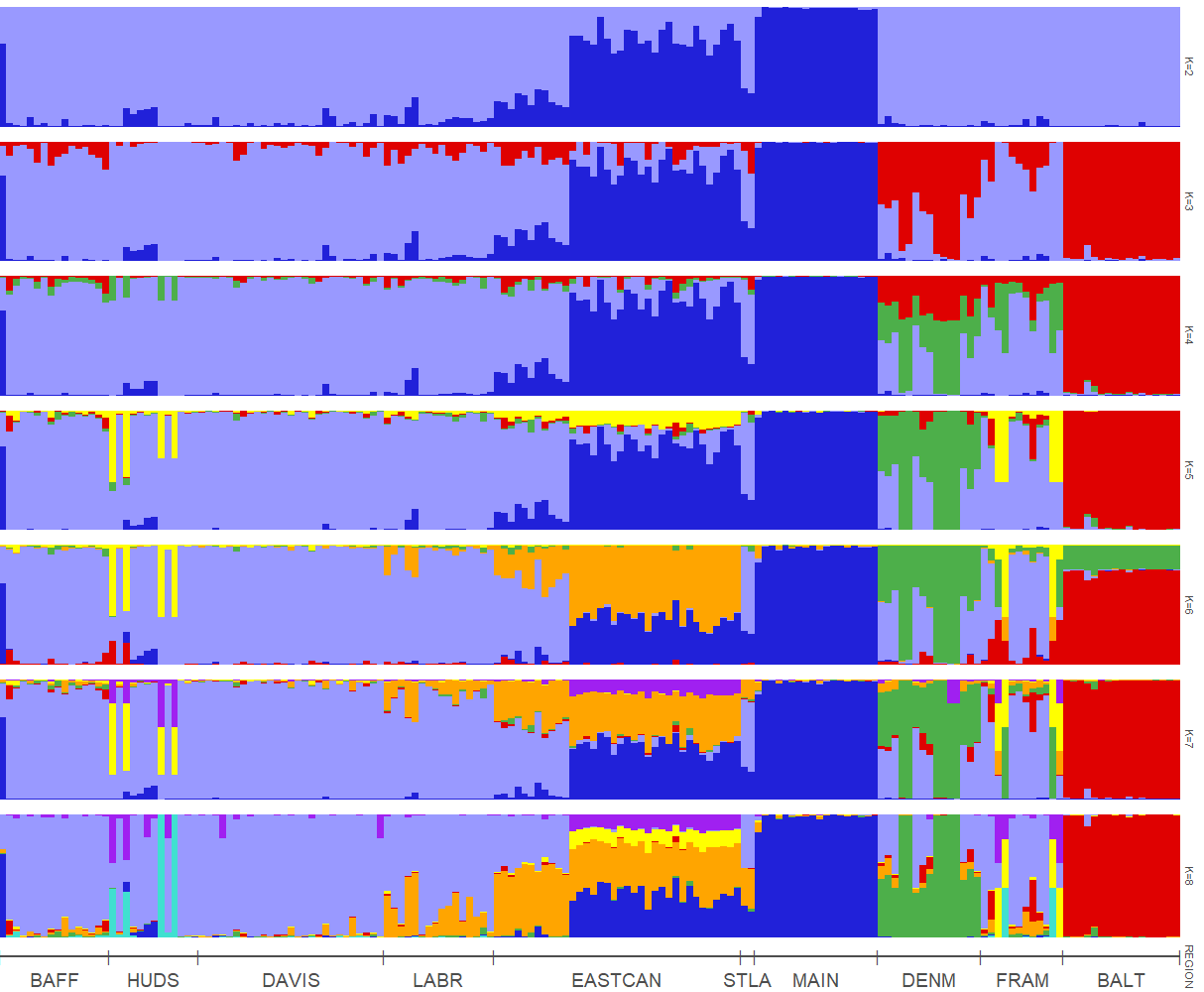


Supplementary Figure S1) Probabilities of assignment of individual guillemots to different genetic populations from STRUCTURE analyses for values of K from 2 to 8. See Table 1 for population abbreviations.
