# Supplementary material for: Population Genomic Structure and Demographic History of Black Guillemots Breeding Across the North Atlantic
Source: Ecol Evol. 2026 Feb 24;16(2):e73126. doi: 10.1002/ece3.73126 (PMC12930286; doi:10.1002/ece3.73126)
Supplement: Supplementary file 2 — Table S1: Information on parameters used for each step of data filtering and the number of SNPs and Individuals that remained following each step. ‘x’ indicates that this parameter was not used for the specified dataset. [file ECE3-16-e73126-s001.docx]

|  |  | **Dataset 1** | | | **Dataset 2** | | | **Dataset 3** | | |
| --- | --- | --- | --- | --- | --- | --- | --- | --- | --- | --- |
|  |  | **Parameter** | **SNPs** | **Individuals** | **Parameter** | **SNPs** | **Individuals** | **Parameter** | **SNPs** | **Individuals** |
| *STACKS populations* | *--min_maf* | 0.01 | 89451 | 232 | 0.01 | 89451 | 232 | x | 95482 | 232 |
|  | *--max_obs_het* | 0.75 |  |  | 0.75 |  |  | 0.75 |  |  |
|  | *--write_single_snp* | Yes |  |  | Yes |  |  | Yes |  |  |
|  | *-R* | x |  |  | x |  |  | x |  |  |
| VCFtools | *--min-meanDP* | 5 | 16778 | 232 | 5 | 16778 | 232 | 5 | 21136 | 232 |
|  | *--max-meanDP* | 120 | 12250 | 232 | 120 | 12250 | 232 | 120 | 15318 | 232 |
|  | *--max-missing* | x |  |  | x |  |  | x |  |  |
|  | ind w missing data | x |  |  | x |  |  | x |  |  |
|  | *--max-missing* | 0.8 | 3834 | 232 | 0.8 | 3834 | 232 | 0.8 | 6444 | 232 |
|  | *--thin* | x |  |  | x |  |  | x |  |  |
|  | ind w missing data | 0.2 | 3834 | 182 | 0.2 | 3834 | 182 | 0.2 | 6444 | 188 |
| Discordance check and duplicate removal |  |  | 3834 | 172 |  | 3834 | 172 |  | 6444 | 178 |
| VCFtools: Hardy-Weinberg Equilibrium | *--hwe* | 0.05 | **3834** | **172** | 0.05 | 3834 | 172 | 0.05 | **6444** | **178** |
| PLINK pruning | *--indep-pairwise* | x |  |  | 50 10 0.2 | **3724** | **172** | x |  |  |

Supplementary Table S1) Information on parameters used for each step of data filtering and the number of SNPs and Individuals that remained following each step. ‘x’ indicates that this parameter was not used for the specified dataset.
